# The impact of a nurse navigation program on health, economic and medical outcomes of cancer patients in Saxony – a multicenter, prospective, randomized controlled trial

**DOI:** 10.1007/s43999-026-00099-z

**Published:** 2026-10-14

**Authors:** Ralf Porzig, Thomas Heckmann, Jens Schnabel, Detlef Quietzsch, Christine König, Daniela Adolf, Ursula G. Froster

**Affiliations:** 1 Sächsische Krebsgesellschaft e.V., Zwickau, Germany; 2 DRK Gemeinnütziges Krankenhaus GmbH Sachsen, DRK Krankenhaus Chemnitz-Rabenstein, Chemnitz, Germany; 3 Facharzt für Innere Medizin / Hämatologie – Internistische Onkologie, Chemnitz, Germany; 4 MSD Sharp & Dohme GmbH, München, Germany; 5 StatConsult GmbH Magdeburg, Magdeburg, Germany; 6 Institut für angewandte Humangenetik u. Onkogenetik Professor Froster, Zwenkau, Germany

**Keywords:** Oncology, Nurse navigator, Patient navigation, Healthcare costs, Health outcomes, Patient-reported outcome

## Abstract

**Background:**

Cancer patients frequently experience complex psychosocial and informational needs that are challenging to address within fragmented healthcare environments, resulting in care inefficiencies and patient distress. Nurse navigation programs aim to provide structured, continuous coordination. This randomized controlled trial evaluated the effect of a nurse navigator program (“Onkolotse”) on hospitalization rates, health-related quality of life, psychological burden, and direct hospital costs in Saxony, Germany.

**Methods:**

In a prospective, multicenter, randomized controlled parallel-group trial across six certified hospital centers, 258 adult cancer patients were randomly allocated (1:1) to an intervention group receiving 12 months of nurse navigator support or a control group receiving standard guideline-based care. The primary endpoint was the 12-month hospitalization rate (≥ 1 additional inpatient stay with at least one overnight stay). Sensitivity analyses evaluated complete cases and alternative deterministic assumptions for missing hospitalization data. Secondary endpoints included anxiety and depression (HADS-D). Exploratory endpoints included health literacy (HLS-EU-Q16), health-related quality of life (SF-36), social support (MOS-SSS), and hospital costs documented by hospital medical controlling.

**Results:**

Between October 2017 and December 2022, 258 patients were randomized (intervention: *n* = 132; control: *n* = 126). The trial was stopped prematurely following an interim analysis due to slow accrual. In the primary ITT analysis (assuming missing follow-ups as non-hospitalized), the hospitalization rate was 43.9% (58/132; 95% CI [35.3%, 52.8%]) in the intervention group versus 51.6% (65/126; 95% CI [42.5%, 60.6%]) in the control group (odds ratio [OR] = 0.74, 95% CI [0.45, 1.20]; *p* = 0.219). In the complete-case analysis (*n* = 192), hospitalization rates were 61.1% (58/95; 95% CI [50.5%, 70.9%]) in the intervention group and 67.0% (65/97; 95% CI [56.7%, 76.2%]) in the control group (OR = 0.77, 95% CI [0.43, 1.40]; *p* = 0.390). Between-group differences in 12-month HADS-D anxiety change (-0.75, 95% CI [-1.65, 0.15]; *p* = 0.103) and depression change (-0.56, 95% CI [-1.47, 0.35]; *p* = 0.225) were not statistically significant. Inpatient hospital costs did not differ significantly between groups (median: 14.224 Euro vs. 12.560 Euro; *p* = 0.136).

**Conclusion:**

The nurse navigator intervention did not demonstrate statistically significant reductions in hospitalization rates, psychological burden, or hospital costs in this underpowered trial. Nevertheless, high patient and physician satisfaction and descriptive outcome patterns indicate that nurse navigation may be a relevant supportive care approach for further evaluation in adequately powered studies using comprehensive healthcare utilization or claims data.

**Trial registration:**

Trial registry name: German Clinical Trials Register (DRKS). Registration number: DRKS00013199. URL: https://www.drks.de/search/de/trial/DRKS00013199/details. Date of registration: 27 October 2017. Furthermore, the study design was published prior to the start of patient enrollment. This took place at the 16th German Congress for Health Services Research (DKVF) on September 26, 2017 [26].

## Introduction

The global incidence of oncological diseases continues to rise [1, 2], placing growing demands on health services. While guideline-based oncological treatments have improved clinical outcomes, cross-sectoral transitions between acute inpatient oncology units, outpatient specialty care, and primary care physicians present structural challenges. Despite numerous support services, patients frequently report difficulties navigating health and social support services, leading to unmet psychosocial needs and uncoordinated care transitions [3–8, 15].

Nurse navigation programs provide individualized support, potentially reducing burdens on both patients and the healthcare system [8–13]. Since 2008, the German Federal Ministry of Health, alongside key cancer organizations, have pursued patient-oriented care through the National Cancer Plan [14]. This plan emphasizes patient health literacy, improved communication, and accessibility to quality information. The IGES report examined structured patient-navigation approaches within the German healthcare system [16].

Regional pilot projects such as OSCAR, PIKKO, HeiMeKOM, and Bosch BKK have demonstrated success [17–20]. The OSCAR program significantly improved cancer-specific quality of life, reduced fatigue and insomnia, enhanced health literacy and physician-patient communication, and was cost-effective. In Saarland, the PIKKO program improved therapy coordination through early needs identification and targeted referral, while HeiMeKom significantly improved patients’ informational orientation and was subsequently integrated into routine care under § 140a SGB V. Similarly, the Bosch BKK support program reduced anxiety, promoted health-conscious behaviour, and improved care coordination, with initial costs offset over time by greater efficiency [19, 20, 30, 31].

Despite positive cost-benefit analyses and interest from policymakers and insurers, nationwide implementation in Germany has been limited [14, 16–21]. Although patient navigation programs are widely established in the United States, structural differences between healthcare systems limit direct comparisons [9, 12, 22]. Recent systematic reviews by Chan RJ et al. [27] and Chen M et al. [28] demonstrate that patient navigation optimizes oncological care by improving access to screenings and significantly shortening the time to treatment initiation. By increasing therapy adherence and improving quality of life, navigation not only enhances patient satisfaction but also reduces readmission rates. Additionally, it proves to be an effective instrument for reducing social disparities and demonstrates high cost-effectiveness, particularly in early detection programs.

In Saxony, a qualification curriculum for Nurse Navigators was established in September 2010 by the Saxony Cancer Society with support from the Saxon State Ministry of Social Affairs and Consumer Protection and the European Social Fund. The training program comprises 130 teaching units and covers a wide range of topics that are essential for supporting cancer patients.

The training program includes:

- Psycho-oncology: Participants learn to recognize high-stress situations, manage patients’ health and identify potential risks, problems and challenges.
- Communication: This area focuses on strategies for improving communication with patients.
- Coaching: Nurse Navigators receive training in decision support, decision assistance and problem solving.
- Exercise and nutrition with cancer: This element of the program educates participants about the importance of exercise and nutrition for cancer patients.
- Health literacy: The training program aims to strengthen the health literacy of patients.
- Participation and being informed: Trainees learn how to encourage patients to be active participants in their treatment and to make informed decisions.
- Help with decision-making conflicts: Nurse Navigators provide support with decision-making conflicts that may arise during treatment.

Currently, over 360 nurses and medical professionals across Germany have completed additional training as Nurse Navigators. They are active in all federal states except Bremen, with approximately one-third based in Saxony. All nurse navigators participating in the trial had completed the specific advanced training and had at least two years of experience in oncological nursing.

The activities of the nurse navigators during the trial were based on a structured intervention protocol that ensures harmonized, cross-sectoral support for cancer patients over a 12-month period. Following a 1:1 randomization, the process began with a timely initial interview immediately after study enrollment. For optimal preparation, the treating physicians informed the nursing staff in advance about diagnoses and therapy decisions, enabling the nurse navigators to provide well-founded support as coaches during the coping process. A primary focus was placed on improving orientation within the healthcare system and promoting individual health literacy [24]. The intensity of the intervention was defined by 18 to 22 contacts per year, with the frequency gradually decreasing during the second half of the year to encourage patient independence. While communication could take place flexibly via various channels, face-to-face meetings after six and twelve months were prioritized to ensure the quality of data collection. Medical follow-up care and specific care in a certified cancer center (including access to social workers) remained in accordance with current guidelines and was identical for both study groups. Quality assurance was maintained through comprehensive documentation following standardized procedures, as well as a concurrent evaluation at three specific measurement points.

The Nurse Navigator program, developed by the Saxony Cancer Society, provides structured guidance for cancer patients.

This study evaluated the Onkolotse program with hospitalization rates as the primary endpoint and psychological burden as the secondary endpoint. Exploratory outcomes included hospital costs, patient-reported outcomes, health literacy, and program satisfaction. The study protocol was previously published [23, 26].

## Methods

### Study design

This was a prospective, randomized, controlled, parallel-group, multicenter superiority trial in which patients were randomly allocated in a 1:1 ratio to an intervention group (receiving Nurse Navigator support) or a control group (standard care). The study spanned 12 months per patient at six centers in Saxony, with data collection at baseline (t1), three months (t2), and 12 months (t3).

The 12-month study period was selected to cover the intensive treatment and early follow-up phase and to capture healthcare utilization and patient-reported outcomes over a clinically relevant period. The 12-month assessment reflects outcomes during the intervention, as nurse navigators supported patients throughout the study period. No post-intervention follow-up beyond the 12-month study period was conducted.

Members of the Patient Advisory Board of the Saxon Cancer Society were involved prior to the set-up and design of this trial.

### Study centers

The study was carried out in six centers: The Gynecological Cancer Center at the German Red Cross (DRK – Deutsches Rotes Kreuz) Hospital in Chemnitz-Rabenstein, the Skin Cancer Center at the DRK Hospital in Chemnitz-Rabenstein, the Colorectal Cancer Center at the Evangelical Diocesan Hospital in Leipzig, the Clinic for General and Visceral Surgery at the Elbland Clinic in Meissen, the Dresden Friedrichstadt Hospital and the Clinic for Gynecology at the St. Georg Clinic in Leipzig. The six participating centers were selected based on the following criteria: (1) certified cancer center with a corresponding minimum number of cases per year, (2) availability of trained nursing staff, and (3) interest and capacity to participate in the study. Each participating study center designated one responsible coordinating physician to oversee local patient screening, clinical documentation, and trial procedures.

### Inclusion criteria

Patients aged 18 years and older who voluntarily participated and signed a consent form were included. Eligibility required at least one inpatient stay in a study center and a diagnosis of a tumor, relapse, or metastases. Doctor approval was also necessary. The study included patients with a number of different cancer types, including gastric cancer, pancreatic carcinoma, colorectal cancer, melanoma and gynecological cancer.

Patients were enrolled within 14 days of diagnosis or hospital admission.

### Exclusion criteria

Patients in palliative or hospice care, those with serious concomitant illnesses requiring priority treatment, participants in disease-management programs, and pregnant or breastfeeding women were excluded.

### Endpoints

#### Primary and secondary endpoint

The primary endpoint was the 12-month hospitalization rate, defined as the proportion of patients with at least one additional hospital admission with a minimum of one overnight stay during the 12-month study period, excluding the index hospitalization for primary cancer therapy.

The primary ITT model applied a deterministic approach in which patients with missing follow-up data were assumed to have had no hospital admissions. Because this deterministic assumption may bias effect estimates when the proportion of missing endpoint data differs between study arms, three sensitivity analyses were a-priori specified:

1. *Complete cases only: Analysis restricted to participants with complete primary-endpoint data (n = 192); for this endpoint*,* this population coincided numerically with the Per-Protocol population*.
2. *Missing both visits imputed as hospitalized*: Patients missing both the 3-month and 12-month follow-ups were classified as hospitalized; otherwise, available visit data were used.
3. *Any missing visit imputed as hospitalized (worst-case)*: Patients missing at least one follow-up visit were classified as hospitalized.

Secondary endpoints comprised changes from baseline in psychological burden assessed via the Hospital Anxiety and Depression Scale (HADS-D anxiety and depression subscales) at 3 and 12 months. Exploratory endpoints included health literacy (HLS-EU-Q16), health-related quality of life (SF-36 Physical and Mental Component Summary scores), social support (MOS-SSS), and patient/physician satisfaction. All secondary and exploratory questionnaire endpoints were evaluated using available cases without imputation.

#### Exploratory endpoints

Exploratory endpoints included: hospital costs submitted to medical controlling after the 3- and 12-month follow-ups (t2 and t3), patient- and health-related outcomes (patient-reported outcomes, PRO), including patient-reported health literacy (HLS-EU-Q16; the European Health Literacy Survey)), social support (MOS-SSS; Medical Outcomes Study Social Support Survey), and physical/mental condition (SF-36 PCS and MCS; Short Form 36 Physical Component Summary and Mental Component Summary).

Program satisfaction was assessed on a four-point scale, from “very satisfied,” to “very dissatisfied.”

Physicians evaluated the program’s usefulness on a 6-point scale from “very useful” to “not useful at all.” They were asked whether they would recommend the program to other physicians (yes/no). Physicians also provided quantitative estimates regarding potential time savings in patient care.

Hospital cost data were obtained from the medical controlling departments of the six participating study hospitals and therefore reflect only costs recorded at the respective study hospitals during the 12-month study period. Costs incurred at non-participating hospitals and costs from outpatient care were not available for this analysis.

### Interim analysis

An interim analysis was conducted in October 2022. The objective—particularly in light of the challenges posed by the COVID-19 pandemic and the fact that the study had been ongoing since late 2017—was to assess whether the primary endpoint had already been achieved or if it could no longer be reached even by continuing the study until the end of follow-up for all planned patients.

### Harms

Due to the non-pharmacological nature of the intervention, no formal assessment of adverse events was performed. However, study participants were asked about their current well-being and any problems at every contact and had the opportunity to report adverse events at any time.

### Sample size calculation

To detect a clinically meaningful difference in hospitalization rates between the control group (25.0%) and the intervention group (12.5%) with 80% statistical power (two-sided α = 0.05, chi-square test), 304 patients were required (normal approximation, PROC POWER SAS 9.4). Considering a drop-out rate of 10%, a total of at least 338 patients (169 per group) were to be included.

### Randomization

Randomization was generated by independent statisticians. Patients were randomly assigned in a 1:1 ratio to an intervention group (receiving Nurse Navigator support) or a control group (standard care). Randomization was stratified by center, cancer type, gender, and age. Within each stratum, a block-wise randomization with random block length (4 or 6) was conducted by StatConsult‘s biometry department. The randomization had been provided via sealed sequentially numbered envelopes to each site at study start, which were opened in the order of patient inclusion. Due to the specific set-up of the study and the type of intervention, the trial was not blinded.

Recruitment of patients was conducted by study physicians. The recruiting staff had no access to the allocation sequence prior to assignment.

Pseudonymized paper questionnaires, completed by patients and physicians, took 30–45 min.

### Statistical analyses

The statistical analyses presented here concentrate on the ITT (intention to treat) population. The analyses were performed with the software SAS 9.4 (SAS Institute Inc., Cary, NC, USA).

#### Primary endpoint

The group difference in hospitalization rates, was examined using chi-square tests at a two-sided significance level of 5% for the main analysis as well as for all sensitivity analyses. A multivariable logistic regression model including treatment group, age, and sex as covariates was fitted for the primary endpoint and each sensitivity analysis.

#### Secondary endpoint

Changes in HADS-D scores were analyzed using t-tests for visit-specific changes from baseline and a mixed model for repeated measures for the longitudinal analysis.

#### Exploratory endpoint

The cost analysis was performed using the Mann-Whitney U test, while other exploratory endpoints were analysed with descriptive statistics.

#### Missing data

Missing data were imputed only for sensitivity analyses of the primary endpoint; otherwise, data were analysed as observed. No formal comparison of baseline characteristics between participants with complete and missing primary-endpoint data was performed.

#### Subgroup and sensitivity analyses

Subgroup analyses on hospitalization rates included descriptive information according to cancer type. No sensitivity analyses were planned in the original study protocol; these were subsequently pre-specified in the Statistical Analysis Plan (internal document).

### Ethics vote

The Ethics Committee of the Saxony State Medical Board approved the study material at its meetings on August 21, 2017 and September 11, 2017 under file number EK-BR-51/17–1 and the amendment on February 21, 2022 under file number EK-BR-51/17 − 2. This ethical approval covers all of the study sites that are used in this study. All patients provided informed consent prior to participation.

## Results

Patient recruitment was conducted between 16 October 2017 and 31 December 2022 across six certified hospital centers. The original recruitment period was planned for 24 months, from October 2017 to October 2019. The final patient visit occurred on 18 January 2023. Annual accrual was 11 patients in 2017, 62 in 2018, 74 in 2019, 46 in 2020, 38 in 2021, and 27 in 2022. Thus, 147 of the planned 338 participants had been enrolled by the end of 2019. Recruitment was slower than anticipated before the COVID-19 pandemic, and pandemic-related infection-control measures and clinical resource constraints subsequently further affected recruitment and follow-up data collection. Following an interim review in October 2022, recruitment was discontinued on 31 December 2022 before the planned sample size of 338 patients was reached.

**Fig. 1:**
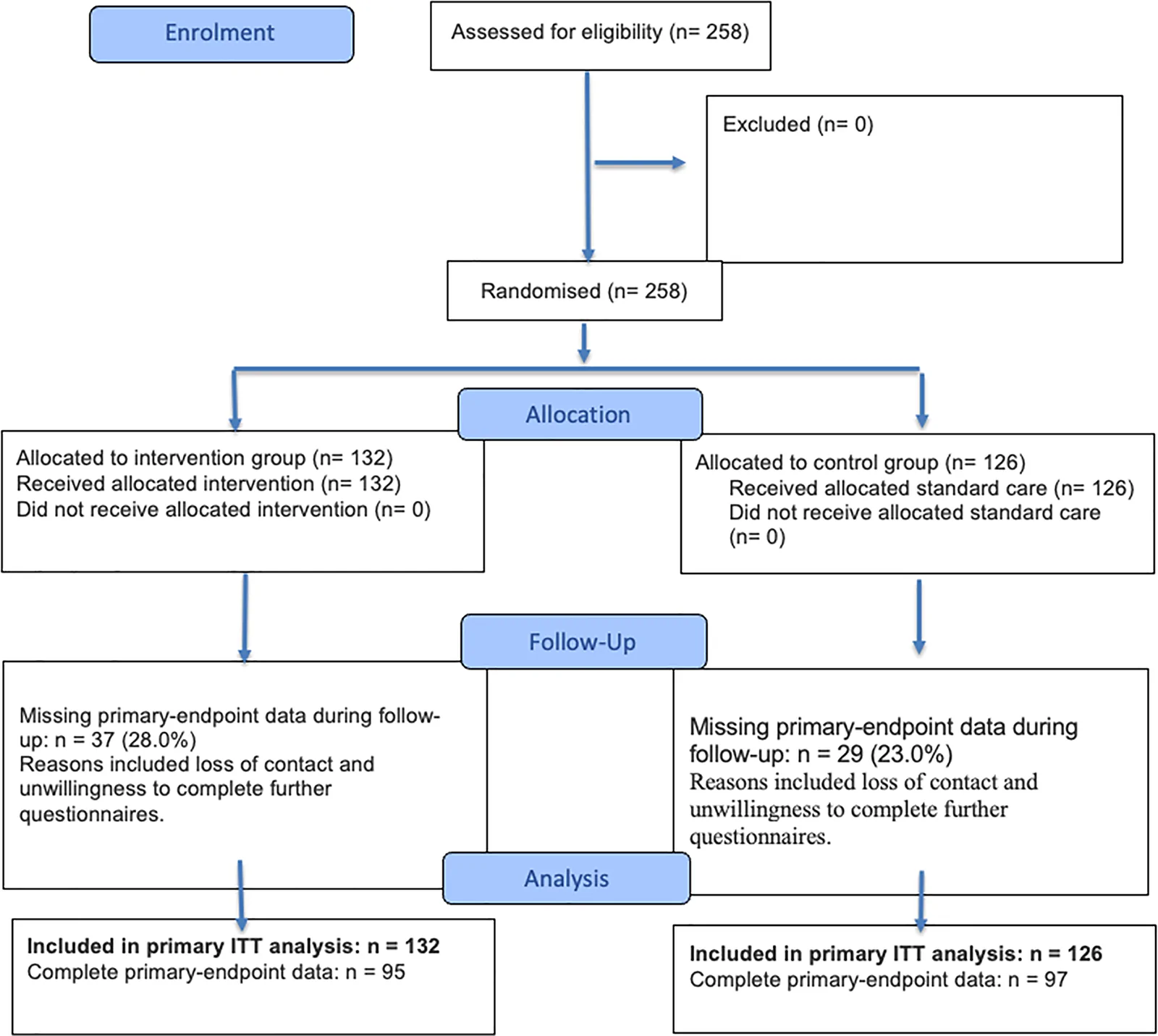
CONSORT 2025 flow diagram [29]. Flow diagram of the progress through the phases of the randomised trial of two groups

A total of 258 patients were randomized (intervention: *n* = 132; control: *n* = 126; ITT population; Fig. 1). Sixty-six patients (25.6%) had missing primary-endpoint data during follow-up (intervention: *n* = 37 [28.0%]; control: *n* = 29 [23.0%]).

The study physicians who enrolled the patients (Table 1) were the attending chief physicians of the respective cancer centers. In addition, outpatient physicians who continued to provide care for the patients within the cancer centers’ networks were also involved.

**Table 1:** Key patient characteristics

Characteristic	Intervention group (*n* = 132)	Control group (*n* = 126)
Age, years - mean (SD)	61.8 (14.0)	62.9 (12.8)
Sex, female - n (%)*	77 (63.6%)	74 (64.3%)
Sex, male - n (%)*	44 (36.4%)	41 (35.7%)
Employment status = employed	42.2%	42.5%
Care degree available	5.2%	5.4%
Colon cancer	35.6%	36.0%
Stomach cancer	4.5%	4.0%
Pancreatic cancer	3.0%	0.8%
Melanoma	19.7%	22.4%
Gynecological cancer	37.1%	36.8%
Cancer stage 0	1.5%	1.6%
Cancer stage I	37.4%	44.8%
Cancer Stage II	15.2%	13.6%
Cancer stage III	19.1%	19.2%
Cancer stage IV	16.8%	11.2%

* Data are presented as n (%) based on available non-missing cases (Intervention group: *n* = 121; Control group: *n* = 115). Sex information was missing for 11 patients in each group, which accounts for the difference from the total randomized population (*n* = 132 and *n* = 126, respectively). Age data were available for 112 participants in the intervention group and 109 participants in the control group. Sex data were available for 121 and 115 participants, respectively. Stage was unknown or not documented in 10.0% (intervention) and 9.6% (control) of patients

### Primary endpoint

In the primary ITT model, assuming no hospitalization for missing follow-up data, 43.9% (58/132; 95% CI [35.3%, 52.8%]) of patients in the intervention group and 51.6% (65/126; 95% CI [42.5%, 60.6%]) in the control group had at least one hospital admission (OR = 0.74, 95% CI [0.45, 1.20]; *p* = 0.219; Table 2). In a multivariable logistic model adjusting for patient age and sex, the difference remained non-significant (adjusted rates: 44.1% vs. 51.4%; *p* = 0.248).

None of the sensitivity analyses showed a statistically significant between-group difference (Table 2). The numerical direction of the group difference varied across missing-data assumptions. In the complete-case analysis (*n* = 192), hospitalization occurred in 61.1% (58/95) of intervention patients and 67.0% (65/97) of control patients (OR = 0.77, 95% CI [0.43, 1.40]; *p* = 0.390; adjusted *p* = 0.500). Imputing missing data as hospital admissions resulted in rates of 72.0% vs. 74.6% (Sensitivity Analysis 2; *p* = 0.633) and 83.3% vs. 81.7% (Sensitivity Analysis 3; *p* = 0.737).

**Table 2:** Hospitalization rate over 12 months across primary and sensitivity analysis models (ITT Population, *N* = 258)

Analysis Model & Imputation Strategy	Intervention Group (*n* = 132)	Control Group (*n* = 126)	Odds Ratio [95% CI]	X^2^ test *p*-value	Multivariable Logistic Regression *p*-value*
Primary ITT Model (Missing follow-ups assumed not hospitalized)	58 / 132 (43.9%) [35.3%, 52.8%]	65 / 126 (51.6%) [42.5%, 60.6%]	0.74 [0.45, 1.20]	0.219	0.248
**Sensitivity Analysis 1** *(Complete cases only / Per-Protocol*,*N = 192)*	58 / 95 (61.1%) [50.5%, 70.9%]	65 / 97 (67.0%) [56.7%, 76.2%]	0.77 [0.43, 1.40]	0.390	0.500
**Sensitivity Analysis 2** *(Missing both follow-ups assumed hospitalized)*	95 / 132 (72.0%) [63.5%, 79.4%]	94 / 126 (74.6%) [66.1%, 81.9%]	0.87 [0.50, 1.53]	0.633	0.637
**Sensitivity Analysis 3** *(Any missing follow-up assumed hospitalized)*	110 / 132 (83.3%) [75.9%, 89.3%]	103 / 126 (81.7%) [73.9%, 88.1%]	1.12 [0.58, 2.14]	0.737	0.667

* To note: Multivariable logistic regression models were adjusted for patient age and sex. If age or sex information was missing in the patient questionnaire, the corresponding information from the physician questionnaire at baseline was used where available. The adjusted primary ITT model and Sensitivity Analyses 2 and 3 included 257 of 258 randomized participants because one participant had missing covariate information. The adjusted complete-case analysis included all 192 participants with complete primary-endpoint data

### Subgroup analysis by cancer type

Colorectal cancer was the most common diagnosis, followed by gynecological tumors, melanoma, gastric cancer, and pancreatic carcinoma. Melanoma and gynecological cancer patients in the Nurse Navigator group showed lower hospitalization rates than controls. No notable differences were observed for colorectal, gastric, or pancreatic cancers (Table 3).

**Table 3:** Descriptive subgroup analysis of hospitalization rate according to cancer type

		Hospitalization rate			
		Estimate		95% CI	
Cancer type	Treatment group	*N*/*N*_total_	%	Lower bound	Upper bound
Colorectal cancer	Intervention	25/47	53.2	38.1	67.9
	Control	24/45	53.3	37.9	68.3
Gastric cancer	Intervention	1/6	16.7	0.4	64.1
	Control	1/5	20.0	0.5	71.6
Pancreatic carcinoma	Intervention	1/4	25.0	0.6	80.6
	Control	1/1	100	2.5	100
Melanoma	Intervention	11/26	42.3	23.4	63.1
	Control	16/28	57.1	37.2	75.5
Gynecological tumors	Intervention	20/49	40.8	27.0	55.8
	Control	23/46	50.0	34.9	65.1

To note: Very small subgroup; 95% CIs are very wide and results are descriptive only

### Secondary endpoint

At 12 months, the between-group difference in change from baseline in HADS-D anxiety was − 0.750 (95% CI [− 1.652, 0.152]; *p* = 0.103). Mean changes from baseline were − 0.926 (95% CI [− 1.599, − 0.253]) in the intervention group and − 0.176 (95% CI [− 0.777, 0.425]) in the control group. For HADS-D depression, the between-group difference in change from baseline was − 0.562 (95% CI [− 1.472, 0.349]; *p* = 0.225).

### Exploratory endpoints

#### Hospital costs

Total hospital costs did not reveal a significant difference between groups (*p* = 0.136); (Table 4).

**Table 4:** Total hospital costs (in Euro)

Treatment group	Total costs in Euros								
	*N*	*N* (miss)	Mean	SD	Min	Q1	Median	Q3	Max
Intervention group	131	1	19805.88	20205.62	0.00	10011.00	14224.07	21351.41	151948.41
Control group	126	0	20283.64	28656.95	0.00	7784.94	12559.53	18421.55	214952.54

#### PRO: health literacy according to HLS-EU-Q16

Mean changes from baseline in HLS-EU-Q16 were 0.8 ± 3.5 versus 0.2 ± 3.2 at 3 months and 1.2 ± 3.6 versus 1.0 ± 3.6 at 12 months in the intervention and control groups, respectively (Table 5).

**Table 5:** Health literacy. Comparison of intervention group and control group over both follow-ups

	Mean change from starting value (t1) ± SD	
Follow-up	Intervention group	Control group
3 months (t2)	*N* = 84	*N* = 93
	0.8 ± 3.5	0.2 ± 3.2
12 months (t3)	*N* = 57	*N* = 77
	1.2 ± 3.6	1.0 ± 3.6

#### Social support according to MOS-SSS

The perceived extent of social support fell throughout the duration of the study. The control group, in particular, perceived a lack of social support more clearly in the second follow-up (Table 6).

**Table 6:** Perceived social support. Comparison of intervention group and control group over both follow-ups

	Mean change from starting value (t1) ± SD	
Follow-up	Intervention group	Control group
3 months (t2)	*N* = 91	*N* = 94
	-2.1 ± 13.1	-1.2 ± 10.8
12 months (t3)	*N* = 61	*N* = 77
	-2.1 ± 12.6	-4.7 ± 15.0

#### Health survey according to SF-36 PCS and MCS

For the SF-36 Mental Component Summary, mean changes from baseline were 4.4 ± 14.1 versus 2.5 ± 11.0 at 3 months and 4.6 ± 12.8 versus 3.4 ± 12.4 at 12 months in the intervention and control groups, respectively. For the Physical Component Summary, the corresponding changes were 2.1 ± 10.4 versus 4.7 ± 9.1 at 3 months and 6.9 ± 11.9 versus 2.6 ± 9.1 at 12 months (Table 7).

**Table 7:** Physical and mental components

SF36	Follow-up	Intervention group	Control group
		Mean change from starting value (t1) ± SD	
Mental Component Summary (MCS)	3 months (t2)	*N* = 77	*N* = 82
		4.4 ± 14.1	2.5 ± 11.0
	12 months (t3)	*N* = 50	*N* = 63
		4.6 ± 12.8	3.4 ± 12.4
Physical Component Summary (PCS)	3 months (t2)	*N* = 77	*N* = 82
		2.1 ± 10.4	4.7 ± 9.1
	12 months (t3)	*N* = 50	*N* = 63
		6.9 ± 11.9	2.6 ± 9.1

Comparison of intervention group and control group over both follow-ups

### Satisfaction with the nurse navigator program (4-point scale)

Patient satisfaction with the Nurse Navigator Program was high, with 60.7% “very satisfied” and 34.4% “rather satisfied.” A majority (95.1%) would recommend the program, citing personal consultations as its most valuable component (68.8%).

Among six treating physicians, two were “very satisfied” and three “rather satisfied,” with all recommending the program.

### Support assessment and time savings

Physicians reported that, in their opinion, 83% of patients received very good or good support. They also estimated that they saved time in 79.1% of cases, particularly in administrative tasks and coordination with other physicians providing treatment.

## Discussion

In this prospective, multicenter randomized controlled trial evaluating a 12-month nurse navigation program (“Onkolotse”) across six certified cancer centers in Saxony, the intervention did not result in a statistically significant reduction in 12-month hospitalization rates, psychological distress, or direct hospital costs compared with standard care. Although between-group differences did not achieve statistical significance, descriptive outcome patterns and exploratory satisfaction assessments were documented for both patients and healthcare providers.

While Nurse Navigator was conducted in Saxony, other regional navigation models have been evaluated in Germany, including OSCAR in North Rhine-Westphalia and Berlin [17], PIKKO in Saarland [18], and HeiMeKOM in Baden-Württemberg [19, 20]. The OSCAR and PIKKO programs similarly utilized nurse navigators qualified under the curriculum developed by the Saxon Cancer Society [17, 18]. However, empirical comparative data on acute hospital readmission rates have not previously been reported for nurse navigation in Germany.

In the pre-specified primary intention-to-treat analysis, participants with missing follow-up information were deterministically classified as not having been hospitalized. This resulted in hospitalization rates of 43.9% (58/132) in the intervention group and 51.6% (65/126) in the control group (OR = 0.74, 95% CI [0.45, 1.20]; *p* = 0.219). Because primary-endpoint data were missing more frequently in the intervention group (37/132; 28.0%) than in the control group (29/126; 23.0%), this deterministic assumption may have biased the comparison in favor of the intervention. In the complete-case analysis, hospitalization rates were 61.1% (58/95) and 67.0% (65/97), respectively (OR = 0.77, 95% CI [0.43, 1.40]; *p* = 0.390). When patients missing both follow-up visits were classified as hospitalized, rates were 72.0% and 74.6% (*p* = 0.633); when any missing follow-up was classified as hospitalization, rates were 83.3% and 81.7% (*p* = 0.737), reversing the numerical direction of the comparison. Thus, none of the analyses demonstrated a statistically significant difference in hospitalization between groups.

The hospitalization rate was selected as the primary endpoint because inpatient treatment represents a major expenditure category in statutory health insurance [32], totaling 111.4 billion Euros (33.1%), which motivated the selection of hospitalization as a health-services endpoint. Health economic evaluations must therefore balance the benefits of new services, such as nurse navigators, against potential savings, for example, through avoided complications.

For the secondary endpoint, anxiety and depression (HADS-D), between-group differences in score changes did not reach statistical significance (adjusted difference in anxiety: -0.75, 95% CI [-1.65, 0.15], *p* = 0.103; depression: -0.56, 95% CI [-1.47, 0.35], *p* = 0.225).

Hospital costs did not differ significantly between groups. Interpretation of this analysis is limited because cost data were restricted to the participating study hospitals and did not capture costs incurred at non-participating hospitals or in outpatient care. In contrast, the OSCAR study reported significantly lower adjusted monthly total healthcare costs for the intervention group (*p* = 0.006) [17], while the PIKKO study found higher inpatient costs in the intervention group [18]. This discrepancy may stem from initial patient allocation, where more intervention group participants in PIKKO required hospitalization from the onset. Given that hospital costs can vary significantly based on cancer type and stage, a more detailed analysis was beyond the scope of this study due to patient numbers.

To ensure accurate data collection regarding patient costs, the registered costs from the study centers’ medical controlling departments were utilized.

Exploratory patient-reported outcomes were analyzed descriptively. At 12 months, mean changes from baseline were 1.2 versus 1.0 points for HLS-EU-Q16, − 2.1 versus − 4.7 points for MOS-SSS, 4.6 versus 3.4 points for the SF-36 Mental Component Summary, and 6.9 versus 2.6 points for the SF-36 Physical Component Summary in the intervention and control groups, respectively. Because these exploratory outcomes were not evaluated as confirmatory endpoints and follow-up data were incomplete, these numerical differences should not be interpreted as evidence of intervention effectiveness.

Perceived social support declined in both groups, though less so in the intervention group. This outcome contrasts with the generally positive patient-reported outcomes. The COVID-19 pandemic may have influenced perceived social support in both study groups; therefore, these exploratory findings should be interpreted cautiously.

Overall, patients in the Onkolotse program highly valued personalized consultations, consistent with findings from the OSCAR study [17]. In PIKKO, participants also rated their satisfaction positively (mean score: 1.96, where 1 represented the highest rating) [18]. Physicians also reported favorable assessments of the program, including perceived time savings in patient care.

Our findings - characterized by statistically non-significant differences in 12-month hospitalization rates and direct hospital costs alongside high patient-reported satisfaction - are consistent with international randomized and transitional care trials on oncology navigation. In a randomized controlled trial across multiple solid tumor entities, Berezowska et al. [33] found no statistically significant effect of hospital-based professional navigation on generic health-related quality of life or acute healthcare utilization, yet patients derived meaningful value in empowerment and self-efficacy, prompting the authors to advocate for risk-targeted rather than broad, unselected enrollment. Similarly, in a cluster-randomized trial of postoperative supportive care navigation for breast and colorectal cancer patients, Sussman et al. [34] observed no measurable reduction in acute readmissions or emergency room presentations, indicating that routine clinical endpoints in unselected cohorts are often dominated by disease trajectory and clinical baseline risk rather than navigation support alone. Furthermore, recent data by Lo et al. [35] demonstrated that while transition-of-care nurse navigation significantly optimizes post-discharge appointment adherence and care continuity, avoiding acute hospital-level events remains largely multifactorial. Taken together, these studies illustrate that effects of nurse navigation on acute healthcare utilization vary across populations, settings, and intervention designs. The present trial does not establish which clinical or patient-reported outcomes are most responsive to nurse navigation. Nevertheless, high patient and physician satisfaction and descriptive outcome patterns indicate that nurse navigation may be a relevant supportive care approach for further evaluation in adequately powered studies using comprehensive healthcare utilization or claims data.

Patients did not report any harmful program effects.

### Influence of the COVID-19 pandemic

The COVID-19 pandemic hindered the study through difficult patient recruitment, delayed data collection, less complete follow-up data and complicated patient contact leading to the necessary transition of patient navigator communication to remote formats. Hospital care structures were severely affected during the pandemic, with staff overburdened and priorities shifting, making face-to-face meetings particularly challenging [25].

In some cases, site-specific regulatory challenges led to the temporary suspension of study activities. Additionally, nurse navigators encountered increasing difficulties in maintaining contact with patients during follow-up, which further complicated study execution and ultimately contributed to its early termination.

More specifically, visiting bans and the prioritization of clinical resources strictly restricted participant access. This required a definitive shift of patient navigator communication to remote formats, which increased the administrative burden on data management and resulted in less complete follow-up data.

The pandemic required greater use of remote contacts and may have affected both intervention delivery and outcome ascertainment. These pandemic-related changes should therefore be considered when interpreting patient-reported outcomes and hospitalization data.

### Supporting the national cancer plan

The intervention is conceptually consistent with the National Cancer Plan’s emphasis on patient orientation and structured support [14]. However, the present study does not provide confirmatory evidence that the intervention improved quality of life or other clinical outcomes.

### Limitations

The study faced several limitations. First, the trial was underpowered due to slow recruitment and disruptions related to the COVID-19 pandemic, leading to premature study termination with 258 of the planned 338 patients enrolled. Consequently, the statistical power was insufficient to confirm small or moderate differences between study arms. Second, hospital cost data were confined to internal billing records provided by the medical controlling departments of the six participating hospital centers. The lack of comprehensive statutory health insurance claims data precluded the capture of admissions to non-study hospital facilities, emergency visits elsewhere, or cross-sectoral outpatient resource utilization.

Third, using the hospitalization rate as the primary endpoint posed methodological challenges. Because follow-up questionnaires only captured hospitalizations occurring within the previous three months, hospital stays occurring between months 3 and 9 were not systematically recorded, introducing a risk of underestimating total hospitalization rates. Missing primary-endpoint data and missing patient-reported outcomes may have introduced attrition bias. For the primary endpoint, this uncertainty was explored using sensitivity analyses specified in SAP v1.0; secondary and exploratory questionnaire outcomes were analyzed using available cases without imputation. Finally, the generalizability of findings is limited, as all participants were recruited from a single German federal state within certified hospital structures.

### Bias

Potential sources of bias include recall bias, as self-reported hospitalizations may be subject to inaccurate patient recollection. Performance bias was also possible due to variability in nurse navigator support. However, standardized oncology nurse training, qualification requirements, and program operating procedures helped mitigate this risk.

### Conclusion and outlook

The Onkolotse intervention did not result in a statistically significant between-group difference in the primary endpoint of hospitalization or in the tested secondary endpoints of anxiety and depression; hospital costs also did not differ significantly between groups. Interpretation is limited by premature termination before the planned sample size was reached, missing primary-endpoint data, incomplete ascertainment of hospitalizations between months 3 and 9, and the restriction of cost data to participating study hospitals. Among respondents, patient and physician satisfaction with the nurse navigation program was high. Further adequately powered studies using comprehensive healthcare utilization and claims data are needed to determine the clinical and economic effects of nurse navigation.

## Declarations

### Data sharing

The cleaned data, the variable description, and the SAS scripts are each available at CRO StatConsult. The data are not publicly available due to data protection regulations (GDPR) and the scope of the participants’ informed consent.
